# Stocking Rate Has No Confounding Effect on the Use of Internal and Inert Markers to Predict Botanical Composition, Diet Quality, Degradability and Passage Rate Kinetics in Sheep

**DOI:** 10.3390/ani10122232

**Published:** 2020-11-27

**Authors:** Bulelani Nangamso Pepeta, Mehluli Moyo, Abubeker Hassen, Ignatius Verla Nsahlai

**Affiliations:** 1 Animal and Poultry Science, School of Agricultural, Earth and Environmental Sciences, University of KwaZulu-Natal, Private Bag X01, Scottsville, Pietermaritzburg 3209, South Africa; bulelani.pepeta@gmail.com (B.N.P.); mehluli.moyo@live.com (M.M.); nsahlaii@ukzn.ac.za (I.V.N.); 2 Department of Animal and Wildlife Sciences, University of Pretoria, Private bag X20, Hatfield, Pretoria 0028, South Africa

**Keywords:** acid insoluble ash, degradability, diet selection, faecal recovery, predict, stocking rate

## Abstract

**Simple Summary:**

Internal markers are components of diets recovered to different degrees in faeces of animals. These feed components maybe helpful in predicting diet selection, nutrient intake and digestibility in animals. The development of sustainable grazing management and practices to prevent the depletion of natural grasslands as a result of overgrazing depends on the knowledge of feed intake, chemical and botanical composition of diets selected and consumed by ruminants. Determination of the botanical composition of diets selected by free ranging ruminants by visual observation is time consuming and tedious, while the use of oesophageal cannulated animals is invasive and impairs animal welfare. Therefore, it is imperative to use non-invasive methods, such as internal markers to predict feed preferences and composition of diets that ruminants consume. However, it is unclear if the accuracy of results obtained using internal markers is confounded by other factors, such as stocking rate, which is expected to determine the availability and botanical composition of pasture, which, in turn, may exert its influence on the diet selected and consumed by grazing animals. The findings of this study, however, showed that stocking rates did not affect diet selection and nutrient intake in sheep, while a combination of internal markers can be used to estimate the quality of diet selected by animals under cafeteria feeding conditions, regardless of the stocking rate used. Thus, the use of modified acid detergent fibre, acid insoluble ash and acid detergent lignin contents in feeds achieved high accuracy and precision in estimating diets selected by sheep. This approach serves as a proof of concept that these markers can be also used in free ranging animals.

**Abstract:**

This study investigated if there is any confounding effect of stocking rate on the use of internal markers to determine and predict the dietary ingredient composition, dry matter intake (DMI) and digestibility of diets consumed by sheep. Fifteen sheep were randomly allocated to stocking rate treatments of one (SR1), two (SR2), four (SR4) and eight (SR8) sheep per pen (space allowance: 31.04 m^2^, 15.52 m^2^, 7.76 m^2^ and 3.88 m^2^ per sheep, respectively) and fed ad libitum maize stover, sorghum stover and veld hay by supplying 110% of previous day’s intake. Sheep were rotated across the treatments in four periods of 10 days. The proportion of feeds selected and total DMI were similar across all stocking rate treatments. However, diets selected by sheep in SR2 had the highest digestibility compared to other treatments. The prediction of the effective degradability of dry matter using acid detergent fibre content achieved an accuracy of 84.6%. A combination of crude protein and neutral detergent fibre contents achieved 63% accuracy in the prediction of the rate of degradation of feeds. The use of acid insoluble ash (AIA) as an internal marker to predict nutrient intake, digestibility, DMI and dietary ingredient intake accounted for 84.3%, 81.2%, 53.0% and 64.1% of the variation, respectively. The predictions of dietary feed proportions and nutrient quality selected obtained with least squares procedure using a combination of modified acid detergent fibre (MADF), acid detergent lignin (ADL) and AIA accounted for 81.0% and 72.4% of the variation, respectively. In conclusion, regardless of the different stocking rate tested in this study, a combination of MADF, ADL and AIA as internal markers can be used to estimate diet and nutrient selection by sheep using the least squares procedure. Hence, these markers can be used to predict ingredient composition of diet, diet and nutrient selection, nutrient intake and digestibility in free ranging animals.

## 1. Introduction

The amount and quality of feed consumed by ruminants are direct determinants of animal performance. The first step in diet formulations that meet animal nutritional requirements for maintenance, growth and production rely on the accurate estimation of the botanical composition of diets selected by animals, as well as feed intake [1]. The per-ingredient composition of selected feed components (diets) is then determined to estimate the nutrient intake derived from such diets. Roughages such as crop residues are common feed resources available to most smallholder farmers, and are utilised by ruminants especially during the dry season when feed is scarce [2,3]. Due to these feedstuffs usually being poor in quality, ruminants feeding on them usually fail to achieve their genetic potential for growth even when fed ad libitum. This is partly because of the deficient nutrient content which include but not limited to: nitrogen, phosphorus and minerals [4], as well as the rumen filling effect of roughages [5]. The slow rates of passage and the low degradability of poor-quality roughages in the rumen restricts voluntary dry matter intake through prolonged retention times and filling effect in the rumen [6] reducing growth performance. Rumen degradability and the rate of passage of feedstuffs become important variables to quantify when measuring digestive aspects of consumed diets. These determine the rate at which nutrients are degraded, and the extent to which nutrients are absorbed by the animal, and ultimately influence intake and animal growth performance [7].

Where basal diet is usually deficient as it is common in most extensive systems, optimal animal productivity is dependent on effective supplementation that aims to meet the nutritional needs of animals. Situation-specific predictions of intake and diet quality will allow farmers to balance the availability of a diet with the required type and quantity of supplements, to achieve the desired production targets [8]. Furthermore, sheep reared in groups experience associated competition for available feed, resulting in differences in diet selected within the herd [9]. This may cause animals lower in the dominance hierarchy to eat less or consume lower quality diets than the average quality of offered diets [10]. Unfortunately, the literature on the impact of stocking rates on the quality and quantity of diets selected and consumed by sheep is scanty. Some studies have been conducted to assess the relationship between animal gain and stocking rate under pasture grazing [11], while the optimum stocking rate beyond which dry matter intake and botanical composition selected by sheep is affected remains unclear. The ability to predict the quantity and composition of feed selected and consumed by ruminants necessitates calculating nutrient intake and degradability potential of diets, which are the key in the simulation of animal performance. A non-invasive alternative or complementary indirect approach to the conventional direct methods of determining the botanical composition, degradability and intake of selected diets is the use of internal markers [12,13]. However, different markers have varying reliability such as recovery rate, among other factors [12]. Nevertheless, the use of internal markers in grazing animals has a great potential, because these markers do not require any dosing of animals and, therefore, avoid disturbance of the normal foraging behaviour of animals [10]. Equally, internal markers do not require time to stabilise in faeces, making it possible to evaluate the short-to-long term grazing responses of animals without disturbing normal foraging behaviour [10].

It is unclear if sheep having the same feed choices, or sheep fed in a group compared to feeding individually, will consume and select diets of the same quantity or quality. Studies should ascertain whether or not such diets will have variation in dietary ingredient selected and intake, or will not differ in quality (digestibility), due to the influence of confounding factors, such as the stocking rate of animals used in a pen during animal nutrition trials. Few studies have used acid insoluble ash, acid detergent lignin and modified acid detergent fibre to predict both botanical composition and diet selection by sheep kept under varying stocking rates. Results from this study would be useful in the planning and design of ruminant nutrition trials. The lack of differences in intake and diet quality selected between individually kept animals and grouped animals would entail grouping animals in feeding trials, rather than keeping animals in individual pens. It was hypothesised that the animal stocking rate would influence the intake, botanical composition and digestibility of the diet selected by sheep and may likely confound the accuracy of the internal markers. This hypothesis was tested using a zero-grazing approach for precise evaluation of predicting dietary parameters under a cafeteria feeding system. The objective of this study, therefore, was to determine and predict the intake, botanical composition and digestibility of diets selected by sheep fed roughages at varying animal stocking rates using internal markers. Regression equations of degradability parameters and chemical constituents of diets were also established.

## 2. Materials and Methods

The study was approved by the Animal Research Ethics Committee of the University of KwaZulu-Natal with approval reference number AREC/030/017M. The trial was conducted at Ukulinga research farm at the University of KwaZulu-Natal, Pietermaritzburg, located at 29°39″ 49, 930′ S 30° 24′ 14,630″ E in the sub-tropical hinterland and at an altitude of approximately 700 m. The area receives an annual rainfall of 735 mm, which falls between October and April and with the average minimum and maximum temperature of 8.9 °C and 25.7 °C, respectively.

### 2.1. Experimental Design and Management of Animals

Fifteen Merino sheep (mean body weight: 46.5 ± 3.3 kg) used in the study were dosed against gastrointestinal parasites using 1.9% (m/v) Albendazole and 99% (m/m) Levamisole hydrochloride before the commencement of the trial. The sheep were housed in pens with dimensions: length, 7.32 m; width, 4.24 m; and area 31.04 m^2^, with concrete floors and iron roof. In a 14-day adaptation period, all sheep were grouped together and fed maize stover (*Zea mays*), sorghum stover (*Sorghum bicolor*) and veld hay (*Themeda triandra*) *ad libitum* with free choice access to clean water. All feeds were milled using a hammer mill to pass through a 12 mm screen (Scientec Hammer mill, serial number 400, Johannesburg, Lab World Pty Ltd., South Africa). Diets were allocated twice a day at 0900 h and 1500 h by providing each diet in separate feeding troughs (dimensions: 610 mm × 390 mm × 210 mm) and placing them at the same time and randomly in the pen daily to avoid conditioned learning of the positions of feeding troughs and dietary ingredients [14]. After the adaptation period, sheep were randomly allocated to animal stocking rates of (i) one sheep per pen (SR1); (ii) two sheep per pen (SR2); (iii) four sheep per pen (SR4); and (iv) eight sheep per pen (SR8), with space allowance of 31.04 m^2^, 15.52 m^2^, 7.76 m^2^, and 3.88 m^2^ per head of sheep, respectively. Sheep were rotated across the four stocking rates in four periods of 10 days each, in a Latin square design such that stocking rates were fixed treatment effects and periods were repeated measures while pens were the experimental units. During each period, sheep were adjusted to faecal bags and new pens for three days followed by seven days for collection of faeces, while feed consumption, spillage and orts were monitored. In each pen, sheep were provided with maize stover (MS), sorghum stover (SS) and veld hay (VH) in separate feeding troughs (dimensions: length 610 mm × width 390 mm × height 210 mm) in a cafeteria style, and the position of feeders were rotated day-to-day. The amount of diets allocated on each day was 110% of the previous day’s consumption. Samples of diets offered and refusals left in feeders were collected daily and bulked for each week and collated across weeks to one bulk sample, from which three samples were taken in each diet for manual separation to determine the proportion of stem and leaves.

The average daily intake from each pen was calculated as the weight of each diet offered minus orts and spillages, divided by the number of animals in the pen. A 10% sub-sample of spillages from each pen was taken and oven-dried at 60 °C for 72 h, and then stored in airtight plastic bags pending manual separation into feed and faecal-contaminant components. Faecal bags were emptied before morning feeding and total faecal output was collected and weighed, while samples were oven-dried at 60 °C for 72 h. From diet consumption and faecal output, total tract digestibility (TTD) of a feed selected by sheep was estimated.

### 2.2. In Sacco Degradability Estimation of Diets

The in sacco degradability of whole diets (SS, MS and VH) as well as fractional components of each diet (leaves and stem) was measured in two fistulated sheep (mean body weight: 58.8 ± 5.0 kg) using the nylon bag technique of Orskov et al. [15], to evaluate the quality of diets without the experimental treatment effect. This was done to determine the potential of predicting the degradability parameters using the standard chemical compositions of roughages. Samples were milled to pass through a 2 mm sieve in an ultra-centrifugal mill (ZM 200, Retsch-Alle 1-5.42781, Haan, Germany), and approximately 3 g of each sample was weighed into ANKOM nylon bags (ANKOM Co, Fairport, NY, USA; internal dimensions: 5 cm × 9 cm; pore size 50 μm), sealed and inserted into the rumen for 3 h, 6 h, 9 h, 24 h, 48 h, 72 h, 96 h and 120 h. Each diet was incubated in triplicates per time interval, and bags were inserted sequentially, such that all bags were removed at the same time to avoid rumen disturbance. Upon removal from the rumen, all bags, including those at zero hour (not incubated in the rumen) were washed with running tap water until the water ran clear and then washed in an automated washing machine for 30 min (6 cycles with each cycle lasting for 5 min). Bags were oven-dried at 60 °C for 72 h and dry matter (DM) disappearance from each bag was calculated. Dry matter disappearance data was fitted into the non-linear model of McDonald [16] to estimate degradation parameters as follows:

(1) $${\mathrm{Deg}\;}_{\mathrm{DM}\left(t\right)}=a+b\;(1-e^{-c\;\left(t\;-\;L\right)})$$

where Deg_DM (t)_ = degradability at time (t), *a* = intercept/rapidly solubilised DM at the beginning of incubation (t = 0; g/kg DM), *b* = slowly degradable fraction of DM (g/kg DM), *t* = incubation time (3 h, 6 h, 9 h, 24 h, 48 h, 72 h, 96 h, and 120 h), *e* = base for natural logarithm, *c* = rate of degradation of the *b* fraction (g/h) and L= lag time (h).

The degradability parameters *a*, *b* and *c* were estimated using non-linear procedures of Statistical Analysis System (SAS), version 9.4 (SAS institute Inc. Carry, NC, USA). Potential degradability of dry matter (PD_DM_) was estimated as follows:

(2) $${\mathrm{PD}}_{\mathrm{DM}\;}=\;a+\;b$$

Effective degradability of dry matter (ED_DM_) was estimated as follows:

(3) $${\mathrm{ED}}_{\mathrm{DM}}=a+\left(\frac{bc}{k}\right)+c$$

where *k* = ruminal outflow rate and calculated to be 0.03/h based on the data reported in the present study under Section 3.2. 

### 2.3. Determination of Passage Rate of Digesta Using Inert Markers

To evaluate the effect of animal stocking rate on passage rate of digesta, one sheep from SR1 and SR2, and two sheep each pair from SR4 and SR8 were selected and dosed with cobalt-ethylenediamine tetraacetic acid (Co-EDTA) and ytterbium markers to determine the passage rate of the liquid and the solid phase of digesta in the rumen and hindgut in each sampling period. The Co-EDTA marker solution was prepared as described by Udén et al. [17] by dissolving sodium-ethylenediaminetetraacetic acid and cobalt chloride in solution, then hydrogen peroxide. The aqueous solution was allowed to stand at room temperature for 4 h to allow crystallisation, after which the crystals formed were filtered, washed with ethanol, oven-dried and subsequently stored until use. Ytterbium-mordanted veld hay (*Themeda triandra*) fibre was used as a marker to assess the passage rate of the particulate (solid) phase of digesta in the rumen and in the hindgut. Ytterbium-mordanted veld hay was prepared following methods by Hatfield et al. [18]. Three hundred and sixty grams (360 g) of veld hay milled using a hammer mill to pass through a 12 mm screen (Scientec hammer mill 400, Lab World Pty Ltd., Johannesburg, South Africa) was soaked in distilled water overnight to remove soluble materials and dried in an oven pre-conditioned to 80 °C for 24 h. Dried veld hay (50 g) was soaked in a freshly prepared solution containing 50g ofytterbium (YbCl_3_.6H_2_O) per litre of distilled water for 120 h. Thereafter, labelled veld hay was strained and rinsed off with distilled water, until the water ran clean to remove unbound ytterbium. The ytterbium-mordanted veld hay was allowed to stand at room temperature overnight to strain all the dripping water. The residue was then oven-dried at 60 °C for 48 h. Dried ytterbium-mordanted veld hay fibre was stored in airtight plastic bags pending administration.

Faecal grab samples were taken from each sheep before the administration of markers (0 h) to determine the baseline concentration of ytterbium and Co-EDTA in faeces. Thereafter, sheep were dosed with 20 g of ytterbium labelled fibre which had been thoroughly mixed with 10 g of lucerne hay (*Medicago sativa*). All animals consumed about 98% of the thoroughly mixed ytterbium-mordanted veld-lucerne hay mixtures. Thereafter, 720 g of Co-EDTA crystals was dissolved in 4320 mL of water and each sheep was drenched with 60 mL of the Co-EDTA solution. Rectal faecal grab samples were taken directly from each sheep by rectal palpation at 3 h, 6 h, 9 h, 12 h, 24 h, 27 h, 30 h, 48 h, 52 h, 55 h, 72 h, 75 h, 78 h, 96 h, 99 h, 120 h, 126 h, 144 h and 168 h after marker administration. All samples were stored inside airtight sample bags and frozen, before subsequent oven-drying at 60 °C for 96 h. Dried samples were ground to pass through a 2 mm sieve in an ultra-centrifugal mill (ZM 200, Retsch-Alle 1-5.42781, Haan, Germany) prior to chemical analyses. Two grams (2 g) of oven-dried faecal samples were weighed into porcelain crucibles and ashed in a muffle furnace at 550 °C for 12 h. Ashed samples were transferred into a 250 mL conical flask and 5 mL of 37% hydrochloric acid was added. The solution was heated to dryness using a block heater in a fume hood. To the dried samples, 5 mL of 6 M nitric acid was added and re-heated to boil and the digested contents were filtered with Whatman filter paper (Whatman plc, Little Chalfont, Buckinghamshire, UK; size: 110 mm) into a 100 mL volumetric flask. The contents left on the edges of the conical flask and the filter paper was washed down the volumetric flask with warm de-ionised water. The content of the volumetric flask was thoroughly homogenised and filled to the mark with de-ionised water. The concentration of cobalt-EDTA and ytterbium markers in these solutions were determined using Inductively Coupled Plasma Optical Emission Spectroscopy (ICP-OES) and the concentration of markers in faeces was calculated subsequently. Marker concentration was fitted into the mathematical model of Grovum and Williams [19] to estimate passage rate parameters as follows:

Y = Ae^−kl (t− TT)^ − Ae^−k2 (t − TT)^ when t > TT and Y = 0 when t < TT (4)

where Y and A are adjustable marker concentrations in the faecal dry matter, k_1_ and k_2_ are rate constants, TT is the transit time (calculated time of the first appearance of the marker in the faeces) and *t* is the sampling time post marker administration. Graphically, marker concentrations in faeces were plotted against time and outliers were eliminated. The natural logarithm (ln) of marker concentration in faeces from the descending slope of the curve was regressed against time, and the gradient (k_1_) and y-intercept (A_1_) of the regression line gave the rate of passage in the rumen. The equation of the regression line from the descending slope was used to estimate the concentration of the ascending portion of the graph by fitting the corresponding sampling times. Estimated values were exponentially raised ($e^{x}$) to generate predicted values from the natural logarithm transformed data. Residual concentrations were calculated as the difference between the predicted concentration values and the actual concentration of the marker. The natural logarithm of the residual marker concentrations was regressed against corresponding times of the ascending portion of the graph to determine the equation of the curve. The gradient (k_2_) and the y-intercept (A_2_) of the residual regression line gave the rate of passage in the hind gut. The two regression lines intersect at point (TT, A), and transit time (TT) was calculated as

TT = (A_2_ − A_1_) / (k_2_ − k_1_) (5)

while mean retention time was calculated as the inverse of the passage rate (1/K). Total tract mean retention time (TTMRT) of liquid and particulate passage rate were calculated as the sum of transit time, rumen and hind gut mean retention times of both liquid and particulate phases [20]:

TTMRT = 1/k_1_ + 1 / k_2_ + TT (6)

### 2.4. Chemical Analysis of Feed and Faecal Samples

Diets, dietary fractions (steam and leaves) were analysed according to AOAC [21] for dry matter (ID 934.01), ash (ID 942.05) and crude protein (ID 968.06) using the LecoTruSpec nitrogen analyser (Leco FP200, LECO, Pretoria, South Africa). Neutral detergent fibre (NDF) and acid detergent fibre (ADF) concentration were determined sequentially on the same sample, using the filter bag technique with an ANKOM 220 fibre analyser [22]. Heat stable amylase and sodium sulphite were used in the NDF assay and the results were expressed inclusive of the residual ash. Hemicellulose was estimated as a difference between NDF and ADF. Acid detergent lignin (ADL) content of samples was equally determined using the ANKOM Technology procedures. Modified acid detergent fibre (MADF) was determined following the method by Collier and Foulds [23], with modifications to accommodate the use of filter bags in an ANKOM 220 fibre analyser. Briefly, one gram of diets and faecal samples in triplicates were weighed into pre-weighed ANKOM fibre bags and thereafter extracted with 2000 mL of acid detergent assay for 2 h. The assay was prepared as 1% *w*/*v* of cethyltrimethylammonium (CTAB) into 0.5 M sulfuric acid in a 2000 mL beaker. Acid insoluble ash (AIA) of plant and faecal samples was determined following procedures described by Van Keulen and Young [24] by subjecting 5 g of milled samples to oven-drying, weighing, boiling in 2 *N* hydrochloric acid, filtration through an ash free Whatman filter paper, drying and ashing.

### 2.5. Prediction ofDiet Composition, Digestibility and IntakeUsing Internal Markers

Faecal recoveries of each marker (MADF, AIA and ADL) were calculated to adjust the concentration of each marker in faeces for incomplete faecal recoveries as the proportion of concentration of each marker in faeces ascending from the consumed feed, as follows:

(7) $$FRM_{i}=\;(F\;\times \;F_{i})\;/\;(A\;\times \;Ai_{\;}+\;B\;\times \;B_{i}\;+\;C\;\times \;C_{i})$$

where *FRM_i_* is the faecal recovery of the marker *i*; *F* is the total faecal output; *F_i_* is the concentration of *i*th marker in faeces; *A, B* and *C* are the amount of feed components in the consumed feed; *A_i_*_,_ *B_i_* and *C_i_* are concentrations of marker *i* in feed components. Botanical composition was obtained by estimating the proportions of feed components (MS, SS and VH) in selected feed, using an optimisation procedure (Solver routine) of Microsoft Excel 2016, version KB4011684, 64-Bit Edition following procedures by the authors [25,26]. The procedure minimises the sum of squared discrepancies between the proportional concentration of a marker (ADL, MADF or AIA) in faeces (A), corrected for incomplete faecal recoveries and the proportional concentration of the marker in feed components (E), as follows:

(8) $$\overset{n}{\underset{i=1}{\sum }}{\left[A-E\right]}^{2}=\overset{3}{\underset{i=1}{\sum }}{\left[\frac{Fi}{Ft}-\frac{a\times D1i+b\times D2i+c\times D3i}{a\times D1t+b\times D2t+c\times D3t}\;\right]}^{2}$$

where *a*, *b* and *c* are the proportions (*p*) of diets D1, D2 and D3 in the feed; F*_i_*, D1*_i_*, D2*_i_* and D3*_i_* are the concentration of the marker in faeces and diets; and F*_t_*, D1*_t_*, D2*_t_* and D3*_t_* are the total concentration of markers in faeces and in components of the feed. Previous work by authors [5,13,26] reported that it is mandatory for the number of internal markers to be greater or equal to the components of the feed. This justifies the use of MADF and ADL which had unsatisfactory recoveries in combination with AIA in this study to predict diet composition with markers (ADL, MADF and AIA) equal to feed components (MS, SS, VH) making *n* = 3. Additionally, the optimisation procedure accounts for incomplete recoveries making the markers suitable for predicting diet composition. The constraints and assumptions used in the Solver routine program include: all three diets were assumed to have equal selectivity (33.3%) and their proportions (*p*) were constrained to be positive and their sum add up to 1 (0 ≤ *p* ≤ 1). The predicted marker concentrations in the selected feed were calculated as the sum of the product of the proportions of feed components and corresponding marker concentrations in components of the feed (a×D1i+b×D2i+c×D3i). The optimiser ran iterations and stopped when the objective cell (sum of the squared differences) had a minimum value at the point at which the concentrations of markers in the diets were the closest to the concentrations of markers in faeces.

In comparison with other evaluated internal markers (MADF and ADL) with low recoveries, AIA with satisfactory recoveries gave the best predictions of intake and digestibility as an indirect technique. Subsequently, intake and digestibility predicted using AIA were compared with direct estimates to determine the accuracy and precision of the predictions. Predicted total dry matter digestibility was calculated from concentration of the marker (AIA) in the feed and faeces (corrected for incomplete faecal reveries), as follows:

(9) $$D\;=\;1-\frac{C_{d}}{C_{f}}$$

where *D* is the predicted total dry matter digestibility, *C_d_* is the concentration of the marker (AIA) in the feed and *C_f_* is the concentration of the marker (AIA) in faeces. Predicted feed intake was calculated from total faecal output and predicted digestibility [27], as follows:

(10) $$I\;=\;\frac{F}{1-D}$$

where *I* is the feed intake, *F* is the total faecal output and *D* is the predicted dry matter digestibility calculated from the concentration of the marker (AIA) in feed components and faeces (corrected for incomplete faecal recoveries). The Kulczynski similarity index was used to evaluate the accuracy of the botanical composition, dry matter intake and digestibility of diet predicted using MADF, ADL and AIA markers [13], as follows:

(11) KSI=100×∑2ci/∑(ai+bi)

where KSI is the Kulczynski similarity index, *ci* is a lesser feed parameter *i* between observed and predicted values and ai+bi is the sum of the observed and predicted values of feed parameter *i*.

Nutrient quality is the proportion of each nutrient (chemical constituent) consumed by the animal from all feed ingredients, whereas nutrient intake is the total amount of each chemical constituents (NDF, ADF, CP and ash) consumed by the animal from all feed ingredients (MS, SS and VH), calculated as follows:

(12) Ni=DIa×nutrient i in a+DIb×nutrient i in b+DIc×nutrient i in c

where *Ni* is the intake of nutrient *i* (NDF, ADF, CP and ash); *a, b* and *c* are dietary ingredients (MS, SS and VH); *DIa*, *DIb* and *DIc* are the dietary ingredient intake of MS, SS and VH.

### 2.6. Statistical Analysis

The Pearson correlation coefficients of chemical constituents of roughages and degradability parameters were established using correlation procedure of SAS 9.4 (SAS institute Inc. Carry, NC, USA). The relationship between the observed and the predicted dry matter intake, dietary ingredient intake, nutrient intake, diet proportion and nutrients selected, and digestibility data were assessed by linear regression analysis. The coefficient of determination (R^2^-value) was used to measure the accuracy of predictions, while root mean square error (RMSE) was used to assess the precision of the predicted values of parameters evaluated. Akaike’s information criterion (AIC) was applied in model selection to measure the relative goodness of fit of the statistical models for predicting degradability parameters from chemical components of diets. The variance inflation factor (*VIF*) was used to assess multicollinearity among prediction input variables, where variables that had high multicollinearity were removed (*VIF* >10). Response in dietary parameters in relation to animal stocking rates was evaluated using the response surface regression procedure.

The mixed procedure of SAS was used to determine the effect of animal stocking rate on dry matter intake, nutrient intake, proportion of feed and nutrients selected, digestibility and passage rate. The effect of period was taken as a repeated measure while grouping of animals in each period was treated as random effect. Initial body weight was taken as a covariate. Differences among the least square means were considered significant at *p* <0.05. The statistical model used was:

Y_ijk_ = µ + S_i_ + P_j_+ A_k_ + ε_ijk_ (13)

where Y_ijk_ = observation (dry matter and nutrient intake, proportion of feed and nutrients selected, digestibility and passage rate), µ = overall mean, S_i_ = effect of animal stocking rate (fixed), P_j_ = period effect (repeated), A_k_ = animal grouping in each period (random) and ε_ijk_ = error term.

## 3. Results

### 3.1. Diet Chemical Composition and In Sacco Degradability

The degradability and chemical composition of whole diets and diet fractions (leaves and stems) were evaluated, in order to establish the correlation relationship between chemical composition and degradability parameters, and to better justify the establishment of prediction equations for degradability parameters from chemical composition. All three diets had low CP ranging from 30.4 to 49.9 g/kg and high NDF ranging from 767 to 893 g/kg, with maize stover having the highest values and veld hay having the least. Except in VH, where stems and leaves had almost similar concentrations of AIA, stems had higher concentration of AIA than leaves in MS (25.7 g/kg vs 13.3 g/kg) and SS (45.4 g/kg vs 16.3 g/kg). Potential degradability (PD_DM_) of forages ranged from 634 to 716 g/kg DM and effective degradability (ED_DM_) ranged from 322 to 414 g/kg DM in Table 1. Correlation coefficients (*r*) of the relationship between chemical composition and degradability parameters of diets are given in Table 2. The portion of dry matter solubilised at the beginning of incubation (*a*) was negatively correlated (*p* <0.05) to both NDF and ADF but tended to be positively correlated (*p* = 0.053) to ED_DM_. The hemicellulose content of feedstuffs was negatively correlated to the rumen degradation rate (*c*) of diets in the rumen *(p* <0.05). The effective degradability of dry matter of diets was equally negatively correlated (*p* < 0.01) to ADF and CP (*p* < 0.05), and positively correlated to the portion of dry matter solubilised at the beginning of incubation (*a*). The predicted degradability parameters from chemical components of diets are reported in Table 3. The NDF (R^2^= 52.5) and ADF (R^2^ = 84.6) were the best single predictors of *a* and ED_DM_, respectively. Similarly, *c* was predicted with ADF and CP as input variables which accounted for 57.2% of the variation. On the other hand, *b* and PD_DM_ were predicted by ADF (R^2^ = 17.4) and NDF (R^2^= 12.1) as single predictors, and accounted for less than 18% of the variation that were regarded as unsatisfactory, respectively.

### 3.2. Effect of Stocking Rate on Diet and Nutrient Intake, Diet Selection, Digestibility and Passage Rate in Sheep

There was a lack of differences (*p* > 0.05) on intake of diets (VH and SS) in relation to stocking rate effect, except for MS, where animals in SR2 had higher intake (*p* < 0.05) compared to other treatments with similar intake. Stocking rate did not affect (*p* > 0.05) total dry matter and ingredient intake in the experimental sheep (Table 4). There was neither linear nor quadratic effect of stocking rate on diet intake, dry matter or nutrient intake in the experimental sheep. Animal stocking rates did not affect the diet selected by the sheep (*p* > 0.05), with both observed and predicted selection for veld hay, maize stover and sorghum stover showing lack of differences (Table 5). Observed values showed that veld hay was mostly selected by sheep in SR4 and SR8, while a nearly equal selectivity could be observed in SR2 and SR1 sheep. The relationships between diet selection, nutrient intake and digesta passage rates in the rumen and hind gut were neither linear nor quadratic. Hence, the quality of diet selected was not affected by stocking rate across the animals. Nevertheless, stocking rate affected the digestibility of selected diets (*p* < 0.05), with sheep in SR2 selecting diets of higher total tract digestibility compared to other stocking rates. Predicted apparent total tract digestibility was similar across all treatments. The predicted and observed value of apparent total tract digestibility corrected for incomplete faecal recoveries of AIA accounted for 81.2% of variation in the adjusted coefficient of determination. The stocking rate did not affect the passage rate of the solid and liquid phase of digesta at both the reticulo-rumen and hind gut (*p* > 0.05) (Table 6). Equally, the rumen mean retention time and total tract mean retention time of sheep was not influenced by the stocking rate. Furthermore, the passage rate, rumen mean retention time, total tract mean retention time showed neither any linear nor quadratic response to the stocking rate.

### 3.3. Effect of Stocking Rate on Faecal Recovery of Markers and Prediction of Diet Components Selected by Sheep

**Figure 1:**
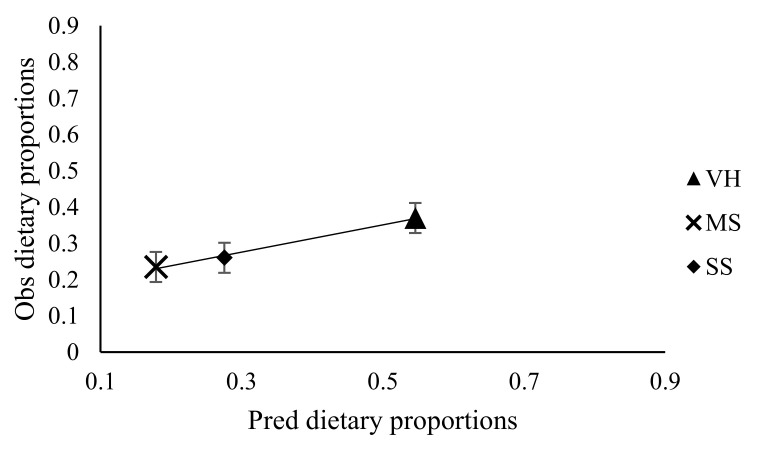
Observed (Obs) versus predicted (Pred) dietary proportions selected by sheep. Regression line y = 1.159 (±0.360)x − 0.0006 (±0.112). RMSE = 0.13; *n* =12 and adjusted R^2^ = 0.45; *p* = 0.01; error bars represent standard error; VH, veld hay; MS, maize stover; SS, Sorghum stover

**Figure 2:**
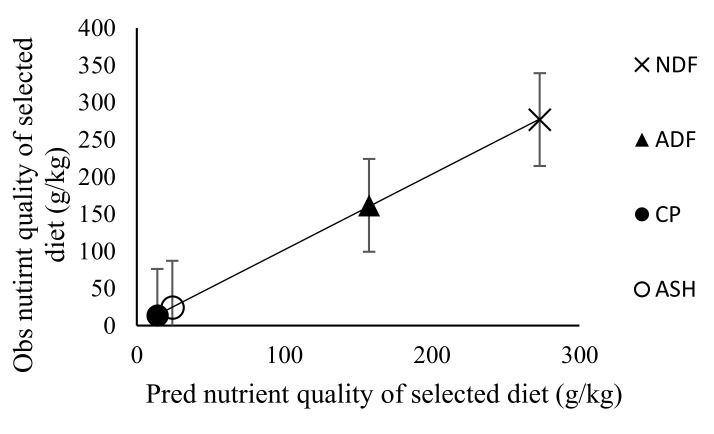
Observed versus predicted (Pred) nutrient quality selected by sheep. Regression line y = 0.984 (±0.007)x − 0.254 (±1.1017). RMSE = 2.9603; *n* = 16 and adjusted R^2^ = 0.999; *p* = 0.001; error bars represent standard error; NDF, neutral detergent fibre; ADF, acid detergent fibre; ASH, ash.

**Figure 3:**
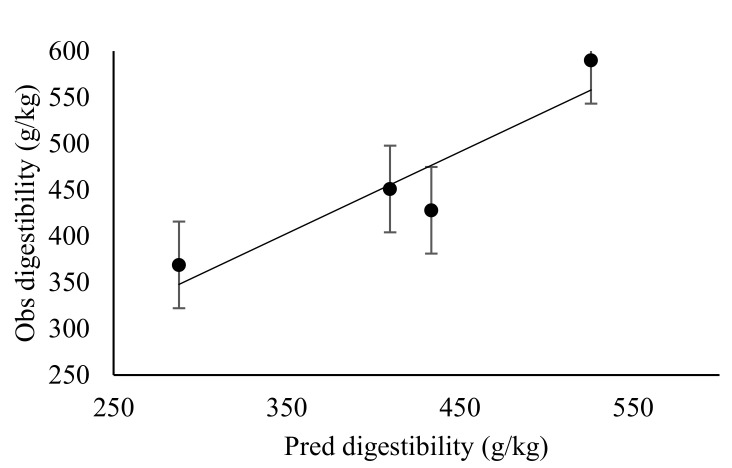
Observed (Obs) versus predicted (Pred) total tract digestibility (g/kg) in sheep. Regression line y = 0.967 (±0.284)x − 29.931 (±132.6014). RMSE = 46.07; *n* = 4 and adjusted R^2^ = 0.78; *p* = 0.08; error bars represent standard error.

**Figure 4:**
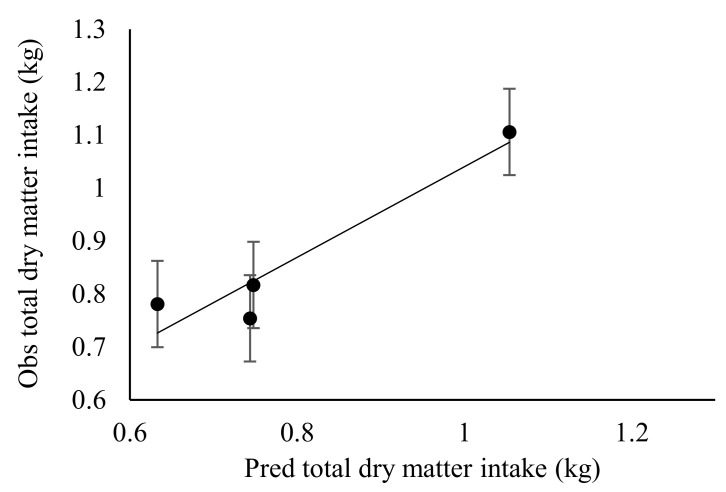
Observed (Obs) versus predicted (Pred) dry matter intake (kg) in sheep. Regression line y = 1.053 (±0.247)x − 0.116 (±0.217). RMSE = 0.0698; *n* = 4 and adjusted R^2^ = 0.851; *p* = 0.0510; error bars represent standard error.

**Figure 5:**
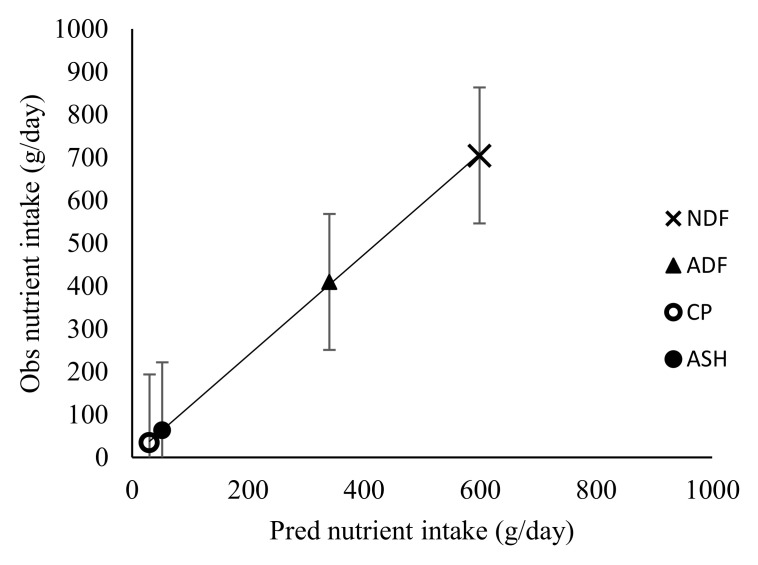
Observed (Obs) versus predicted (Pred) dietary intake by sheep. Regression line y = 0.601 (±0.324)x + 0.0956 (±0.100). RMSE = 0.116; *n* = 12; adjusted R^2^ = 0.182; *p* = 0.0931; error bars represent standard error.

**Figure 6:**
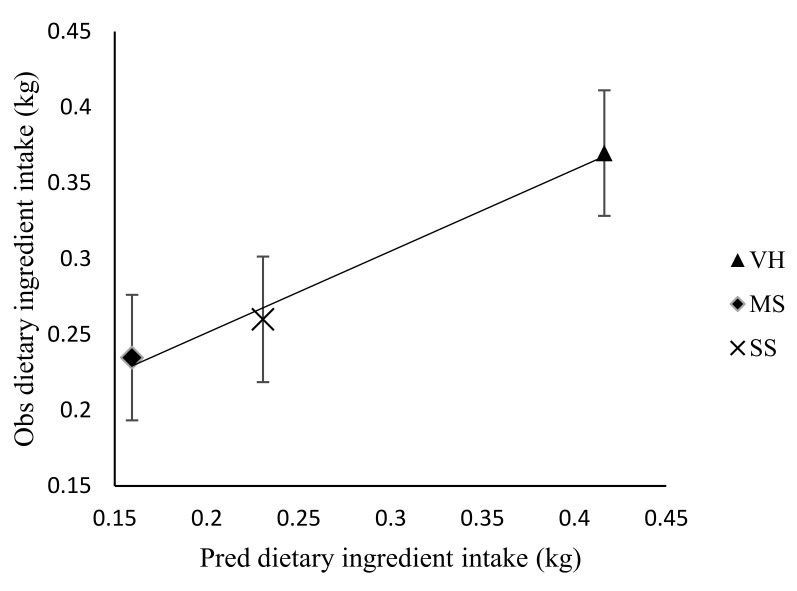
Observed (Obs) versus predicted (Pred) nutrient intake by sheep. Regression line y = 0.797 (±0.05)x + 13.520 (±19.160). RMSE = 52.68; *n* = 16; adjusted R^2^ = 0.925; *p* = 0.0001; error bars represent standard error.

The predictions of dietary proportions were meaningful only when the number of markers was greater or equal to the number of diets included in the least square procedure predictive model. However, marker concentrations in either the leaf or stem portions of diets were lower compared to whole diets. Therefore, only values from whole diets were used in the model for predicting botanical composition. The faecal recoveries (FR) of MADF, ADL and AIA were similar (*p* > 0.05) across the stocking rates (Table 7). There was no linear or quadratic relationship between the FR of markers and stocking rate. The faecal recovery of MADF ranged from 29.3–49.0%, while ADL ranged from 46.2–76.0% and AIA ranged from 96.7–120%. The proportion of stems in refusals ranged from 77.0–86.5% (MS), 76.6–92.3% (SS) and 28.5–65.4% (VH) (Table 8). From the predictions obtained with AIA marker, the variation in the adjusted coefficient of determination in predicting nutrient intake was 84.3% and 53.0% for dry matter intake, while it was 64.1% for dietary ingredient intake. Predictions of dietary proportions and nutrient quality selected using a combination of markers (MADF, ADL and AIA) in the least square optimisation procedure accounted for 81.0% and 72.4% of the variation in adjusted coefficient of determination, respectively. Figure 1, Figure 2, Figure 3, Figure 4, Figure 5 and Figure 6 show the linear regression relationship between the predicted and the observed dry matter intake, dietary ingredient intake, nutrient intake, proportion of feed and nutrients selected and digestibility of diets selected by sheep. There were only few components of diets selected by sheep that had wide variation between the predicted and observed data sets, be it dry matter intake and dietary ingredient intake, with R^2^ values of 0.53 and 0.64, respectively. Whereas, in contrast, dietary proportions and digestibility had an R^2^ value of 0.81, nutrient quality and nutrient intake had R^2^ values of 0.72 and 0.84, respectively.

## 4. Discussion

The findings of the correlation analysis from this study suggest that there are possibilities of estimating degradability parameters (*a*, *b*, *c* and ED_DM_) of a given forage using its chemical composition. Gerber [28] and Moyo et al. [29] applied this principle in the estimation of degradability of protein and dry matter of feeds, respectively. Nsahlai et al. [30] used similar approaches for the estimation of the nitrogen degradable parameters and dry matter degradability from chemical constituents for fresh leaves of multipurpose trees. In our findings, the degradability parameters of roughages were predicted from their chemical components (CP, NDF, ADF and ash) with HEM excluded as one of the input variables, due to an observed linear dependency between the variables and HEM, more so, the variance inflation factor (*VIF*) was greater than ten (*VIF* > 10). Determination of the degradability parameters of forages using the in sacco nylon bag technique is tedious, time consuming and the use of cannulated animals is invasive and impairs animal welfare. Nevertheless, the prediction of degradability parameters from chemical components is cheap, non-invasive, fast and practical, especially to institutions with limited or no resources to conduct in vivo degradability trials. Additionally, the rate and extent at which diets are digested in the rumen are vital determining factors of nutrient utilisation by the ruminants [31].

It is generally believed that cereal straws induce low levels of dry matter intake because of poor digestibility, due to their inherently high fibre with low nitrogen content. In most livestock systems, the most limiting nutrient which influences animal performance is usually protein [32] and, therefore, animals are expected to select diets that adequately provide this nutrient. Results from the current study, however, indicate that these diets had low CP (<80 g/kg). Equally, it appears the variability in the CP contents among the diets may not be significant enough to warrant such selection. Housed animals in a cafeteria feeding style alter diet consumption by adjusting the proportions of diets consumed among the offered choices while maintaining the same overall total dry matter intake. With grazing animals, an increase in stocking rate is expected to increase competition for the same feed, especially where selectivity and choice are limited [33]. For housed ruminants, an interplay of competition and association, where visual cues of one sheep consuming a diet encourage others to feed on the same diet likely exists, influencing feeding patterns [32,34]. This is observable with sheep in SR2 (space allowance of 15.52 m^2^ per head of sheep) exhibiting higher digestibility, compared to sheep on SR4 and SR8, which had a higher stocking rate (space allowance of 7.76 m^2^, and 3.88 m^2^ per head of sheep, respectively), or the SR1, where sheep were penned individually. Nevertheless, this difference in diet quality consumed did not reflect in the selectivity of the various diets on offer. Equally, fewer diet options, as well as a higher stocking rate, have been reported to reduce diet selection in ruminants [35]. Where the few diets on offer have close chemical characteristics, stocking rates may not significantly influence diet selection in housed animals. Previous feed experience has also been noted to effect diet selection and intake [36]. Sheep on this trial had previous feeding experience with VH, as they were reared from an early age under VH, and this may also account for the relatively higher intake of VH, despite being lower in CP and higher in NDF compared to SS and MS.

The observed apparent total tract digestibility in SR2 is indicative of the impact of facilitation of feeding and competition, which both had a little or no effect on consumption of MS, a diet with the highest protein content. The associative effect of high crude protein content of selected diet (MS) on digestibility is related to increased proliferation of microbial population, resulting in higher digestibility. Animals in SR2 had a space allowance of 15.52 m^2^ per head of sheep and the animals were a pair, which may have reduced the effect of competition as compared to SR4 and SR8. Therefore, animals selected a diet with better crude protein content. Generally, the availability of companions in group-fed animals increases activity and visual cues, which stimulate eating unlike animals penned in isolation. This can be inferred to the observed digestibility in SR2 compare to SR1. However, the facilitated feeding behaviour that would have likely caused an increase in feed intake in lambs in the study of Odoi and Owen [34] was not observed in sheep in the present study. Competition for feed would be expected to be high for the sheep kept at a high stocking rate of eight sheep per pen, likely reducing time spent eating and feed intake [10,32]. Given that feed intake was not affected by stocking rates in this study, sheep at a high stocking rate may have increased eating rates and bite size to achieve adequate intake. This phenomenon has been reported by Ruckstuhl [37]. In ruminants, feed intake is noted to be a product of bite-size and biting rate [38]. While diet quality is expected to enhance selection and intake, competition is noted as capable of also affecting bite-size and biting rate. In the study by Ruckstuhl [37], there was no correlation between bite rates and the quality of diets available. In that study, sheep were able to adjust their bite rates to accommodate time spent for alertness of predation and competition association within the herd to meet adequate feeding requirements. Therefore, this partly justifies the possibility that animals in SR4 and SR8 of the current study manifested similar behaviour of increasing the bite rates or size to achieve similar dry matter intake to other animal stocking rates. However, our results fall short of being conclusive, and this calls for research on whether the dry matter intake was controlled using either bite rates or bite size.

It is possible that in the attempt to compensate for high competition, due to the increased number of animals per pen, sheep in SR8 selected a higher proportion of VH. Such compensation is likely motivated by the reticulo-rumen filling effect of veld hay owing to its high DM and low CP content compared to SS and MS. Sheep can eat little amounts of VH and have their reticulo-rumen filled for a longer period, rather than leaves from SS and MS that have low biomass, which was evident in sheep selecting more leaves than stems of SS and MS diets (Table 8). The adoption of this selection behaviour was likely linked to attainment of satiety. Illius and Gordon [39] reported a similar response, where large grazing animals selected grasses in large proportions in their diet relative to forbs and shrubs, because of the high biomass and reticulo-rumen filling effect of grasses compared to shrubs and forbs.

There was a lack of significance in both the linear and quadratic relationships between stocking rate and diet selection, intake and passage rate in this study. These findings suggest that the minimum stocking rate that would have negative effects on diet selection, intake and passage rates occur at stocking rates higher than eight sheep per 31.04 m^2^ or at a space allowance lower than 3.88 m^2^ per head of sheep.

Faecal recovery rates (FR) of markers in nutritional studies are the true reflection of the effectiveness of the markers and allow the establishment of representative relative comparison of markers in estimating dietary parameters in question [26]. Faecal recoveries of internal markers in this study were similar to Huhtanen et al. [40] where AIA had high FR in comparison to ADL. However, in the present study, there was an additional internal marker (MADF) not used in the study by Huhtanen et al. [40], which had the least FR. Low faecal recoveries of MADF (FR < 50%), ADL (FR < 100%) in the present study may be due to a feeding behaviour manifested by sheep of selecting more leaves than stems (Table 8) in their diets, given that stems had higher MADF and ADL contents compared to leaves. Therefore, the amount of marker recovered in faeces was lower than the actual consumed concentration of the marker from selected plant parts. Contrary to the low faecal recoveries of MADF and ADL, AIA had faecal recoveries of ≈100% and beyond. Faecal recoveries of AIA more than 100% in SR1 and SR2 may be due to overestimation as a result of a behaviour observed in sheep licking areas where urination previously occurred, resulting in the consumption of soil and dust particles. Similarly, Huhtanen et al. [40] noted that overestimation of faecal recovery could be due to soil contamination of feed or consumption of spillages contaminated with dust/soils, which may inflate the recoveries of AIA. As expected, the FR rates of markers were not affected by animal stocking rate, because the total faecal outputs were bulked within treatments to reduce the diurnal and individual animal-animal variation in concentration of the markers in faeces. Predicted and observed botanical composition of diets (KSI = 90.90%), total dry matter intake (KSI = 85.54%) and apparent total tract digestibility (KSI = 94.74%) adjusted for incomplete faecal recovery rates had fairly good similarity indices to observed values. The observed low discrepancies in our findings justify that it is not necessary to use situation-specific recoveries (individual animal and grazing time-dependent recoveries) suggested by Ferreira et al. [26], as opposed to mean faecal recoveries of markers (mean recoveries within a treatment pooled across periods) used in the present study. The regression relationship between the observed and predicted dietary proportions and nutrient quality obtained using MADF, ADL and AIA had R^2^ values more than 70%. This suggests the potential use of the combination of the internal markers to estimate botanical composition and, subsequently, nutrient quality, using optimisation procedures that minimise the sum of the squared differences between the marker adjusted for incomplete recoveries in faeces and different mixture of forages. Digestibility, dry matter intake, nutrient intake and dietary ingredient intake predictions with AIA adjusted for incomplete faecal recovery rates had R^2^ values more than 50%. Therefore, these findings suggest that is possible to use AIA in predicting dry matter intake, nutrient intake and dietary ingredient intake in nutritional studies. However, these findings show that dry matter intake should be predicted with careful consideration, because of the low coefficient of determination between the observed and the predicted results.

## 5. Conclusions

There were no differences on the rate of passage, intake and diet selected by sheep as a result of different animal stocking rates tested in this study. Total tract digestibility and intake levels of MS diet were affected by animal stocking rate and were highest in SR2 relative to others. The regression models were able to predict degradability parameters (*a*, *c* and ED_DM_) from chemical components of roughages. Except for dry matter intake, predicted dietary ingredient intake, nutrient intake and digestibility of diet selected by sheep using AIA had good predictions and their predictions were satisfactory. Diet and nutrient selection may be estimated in animals without situation specific faecal recoveries, and these are the fundamental variables in feed formulation and grazing management. Therefore, this study demonstrates that the combination of MADF, ADL and AIA can be used to estimate diet and nutrient selection by sheep using the least squares procedure, regardless of the stocking rate tested. Therefore, future studies on management of grazing animal such as feeding behaviour should be evaluated at stocking rates higher than eight sheep per 31.04 m^2^.

## Figures and Tables

**Table 1:** Chemical composition and degradability parameters of diets offered and diet components.

Diet	^1^ Chemical Composition (g/kg DM)									^2^ Degradability Parameters				
	DM	NDF	ADF	MADF	AIA	ADL	CP	Ash	HEM	*a*	*b*	*c*	PD_DM_	ED_DM_
**Maize stover**	910	893	542	771	17.1	21.1	49.9	41.4	351	101	534	0.02	634	322
**Sorghum stover**	882	877	512	695	26.4	39.0	43.4	110.8	365	118	573	0.02	691	347
**Veld hay**	911	767	430	497	20.1	73.3	30.4	74.6	337	188	528	0.02	716	414
**Maize stover stem**	937	858	545	531	25.7	26.1	52.9	34.4	313	130	583	0.01	713	300
**Maize stover leaves**	921	837	450	578	13.3	13.6	52.2	39.5	387	101	536	0.04	636	393
**Sorghum stover stem**	917	844	464	538	45.4	28.7	43.8	56.0	381	175	580	0.03	754	390
**Sorghum stover leaves**	898	850	472	400	16.3	19.3	40.1	66.4	378	108	513	0.02	621	337
**Veld hay stem**	907	699	419	449	17.4	54.5	31.5	48.2	310	n.d	n.d	n.d	n.d	n.d
**Veld hay leaves**	899	756	460	429	14.7	59.9	32.4	38.5	296	n.d	n.d	n.d	n.d	n.d
**SEM**	4.94	12.4	13.5	38.6	3.13	6.53	2.79	7.67	10.7	12.5	9.93	0.003	17.8	14.7

^1^ DM, dry matter; NDF, neutral detergent fibre; ADF, acid detergent fibre; MADF, modified acid detergent fibre; AIA, acid insoluble ash; ADL, acid detergent lignin; CP, crude protein; HEM, hemicellulose. ^2^ *a*, intercept and rapidly fermentable proportion of DM (g/kg); *b*, slowly degradable fraction of DM (g/kg); *c*, rate of degradation of *b* (h^−1^); PD_DM_, potential degradability (g/kg); ED_DM_, effective ruminal degradability at k = 0.03 per hour (g/kg); n.d, not determined; SEM, standard error of means.

**Table 2:** Pearson correlation coefficients of chemical components and degradability parameters of experimental diets.

Parameter	NDF	ADF	ADL	CP	Ash	HEM	*a*	*b*	*c*	PD_DM_	ED_DM_
**NDF**		0.990 (0.093)	−0.509 (0.660)	0.978 (0.135)	−0.091 (0.942)	0.803 (0.407)	−0.997 (0.048)	0.495 (0.670)	−0.756 (0.455)	−0.809 (0.400)	−0.988 (0.100)
**ADF**	-		−0.628 (0.568)	0.997 (0.043)	−0.234 (0.850)	0.708 (0.500)	−0.998 (0.045)	0.364 (0.763)	−0.653 (0.547)	−0.886 (0.307)	−0.999 (0.008)
**ADL**	-	-		−0.679 (0.525)	0.904 (0.282)	0.106 (0.933)	0.572 (0.613)	0.497 (0.669)	−0.178 (0.885)	0.917 (0.261)	0.637 (0.560)
**CP**	-	-	-		−0.299 (0.807)	0.658 (0.542)	−0.991 (0.088)	0.301 (0.806)	−0.600 (0.590)	−0.915 (0.264)	−0.999 (0.035)
**Ash**	-	-	-	-		0.521 (0.651)	0.165 (0.895)	0.820 (0.388)	−0.584 (0.603)	0.658 (0.543)	0.246 (0.842)
**HEM**	-	-	-	-	-		−0.756 (0.455)	0.916 (0.263)	−0.997 (0.048)	−0.299 (0.807)	−0.699 (0.507)
***a***	-	-	-	-	-	-		−0.429 (0.718)	0.704 (0.502)	0.851 (0.352)	0.997 (0.053)
***b***	-	-	-	-	-	-	-		−0.943 (0.216)	0.110 (0.930)	−0.353 (0.771)
***c***	-	-	-	-	-	-	-	-		0.227 (0.855)	0.644 (0.555)
**PD_DM_**	-	-	-	-	-	-	-	-	-		0.891 (0.300)
**ED_DM_**	-	-	-	-	-	-	-	-	-	-	

NDF, neutral detergent fibre; ADF, acid detergent fibre; ADL, acid detergent lignin; CP, crude protein; HEM, hemicellulose; *a*, intercept and rapidly fermentable proportion of DM (g/kg); *b*, slowly degradable fraction of DM (g/kg); *c*, rate of degradation of *b* (h^−1^); PD_DM_, potential degradability (g/kg); ED_DM_, effective ruminal degradability at k = 0.03/h; (-), removed mirror images; values in brackets represent *p*-values and values in bold are *p*-values < 0.05.

**Table 3:** Predictions of degradability parameters (**y**) from chemical components (**x**) of diets.

^1^ Y	^2^ X	N	Models	AIC	*p*-Value	RMSE	R^2^
***a***	NDF	7	678 (±232.6) − 0.65 (±0.275) NDF	48.8	0.07	27.0	0.525
	CP	7	264 (±68.62) − 2.98 (±1.517) CP	49.0	0.11	29.5	0.435
	NDF, CP	7	580 (±290.7) − 0.46 (±0.412) NDF − 1.34 (±2.085) CP	49.1	0.19	28.8	0.569
	NDF, ADF	7	740 (±285.3) − 0.84 (±0.512) NDF + 0.21 (±0.452) ADF	49.4	0.20	29.4	0.550
	NDF, Ash	7	660 (±260.5) − 0.64 (±0.303) NDF + 0.17 (±0.457) Ash	49.6	0.21	29.7	0.541
***b***	ADF	7	423 (±124.0) + 0.26 (±0.253) ADF	48.4	0.35	28.3	0.174
	CP	7	489 (±67.0) + 1.35 (±1.481) CP	48.0	0.41	28.8	0.142
	CP, Ash	7	420 (±110.1) + 2.27 (±1.913) CP + 0.46 (±0.570) Ash	49.6	0.54	29.9	0.263
	ADF, CP	7	0.07 (±0.026) − 0.0002 (±0.0001) ADF + 0.001(±0.0004) CP	70.0	0.18	0.006	0.572
***c***	NDF, ADF	7	0.002 (±0.060) + 0.0001 (±0.0001) NDF − 0.0002(±0.0001) ADF	69.0	0.24	0.006	0.513
	NDF, CP	7	0.03 (±0.067) + 0.0001 (±0.0001) NDF + 0.0005(±0.0005) CP	69.0	0.33	0.006	0.633
**ED_DM_**	ADF	7	771 (±79.10) − 0.85 (±0.162) ADF	42.1	<0.01	18.0	0.846
	ADF, Ash	7	761 (±94.33) − 0.84 (±0.182) ADF + 0.08 (±0.311) Ash	44.0	0.02	20.0	0.849
	ADF, CP	7	772 (±89.01) − 0.86 (±0.231) ADF + 0.14 (±1.325) CP	44.1	0.02	20.1	0.847
	NDF, ADF	7	780 (±195.3) − 0.02 (±0.350) NDF − 0.83 (±0.309) ADF	44.1	0.02	20.1	0.846
**PD_DM_**	NDF	7	1053 (±449.8) − 0.44 (±0.531) NDF	57.0	0.45	52.3	0.121

^1^*a*, intercept and rapidly fermentable proportion of DM (t = 0); *b*, slowly degradable fraction of DM; *c*, rate of degradation of *b*; PD_DM_, potential degradability (a+b); ED_DM_, effective ruminal degradability (k = 0.03/h); ^2^ NDF, neutral detergent fibre; CP, crude protein; ADF, acid detergent fibre; AIC, Akaike information criterion; RMSE, root mean square error; R^2^, coefficient of determination.

**Table 4:** Effect of animal stocking rate on diet intake, total dry matter and nutrient intake (g/d).

^1^ Diet Intake	Stocking Rate (Sheep per Pen)				^2^ Mixed Model	
	1	2	4	8	SEM	Significance
**Veld hay**	301	306	446	434	70	NS
**Maize stover**	146^b^	416^a^	143^b^	209^b^	62	*
**Sorghum stover**	262	384	163	175	73	NS
**Total DM intake**	709	1111	766	817	171	NS
**FR-total DM intake**	651.4	1000	713.0	737.4	110	NS
**KSI = 85.5%**	-	-	-	-	-	-
**Nutrient intake**						
**CP**	28.3	45.6	31.5	34.5	4.70	NS
**NDF**	591	943	613	672	94.50	NS
**ADF**	343	553	353	389	55.6	NS
**Ash**	49.5	84.8	56.0	62.7	9.13	NS

^1^ FR—total DM intake, predicted total dry matter intake adjusted for incomplete faecal recoveries of acid insoluble ash marker; KSI, Kulczynski similarity index between the overall predicted and the observed total dry matter intake; CP, crude protein; NDF, neutral detergent fibre; ADF, acid detergent fibre; ^2^ SEM, standard error of mean; NS, not significant (*p* > 0.05); * significantly different (*p* < 0.05); ^a,,b^ means with different superscripts within a row differ significantly; (-), omitted values because the KSI is for the overall vales and not for each mean.

**Table 5:** Effect of animal stocking rate on diet selection (g/g), predicted diet selection (g/g), diet quality (g/kg) and apparent total tract digestibility (g/kg DM) in sheep.

^1^ Parameter	Stocking Rate				^2^ Mixed Model	
	1	2	4	8	SEM	Significance
**Diet selection(g/g)**	**Observed**					
**Veld hay**	0.409	0.286	0.632	0.557	0.09	NS
**Maize stover**	0.236	0.369	0.162	0.239	0.07	NS
**Sorghum stover**	0.356	0.346	0.205	0.205	0.09	NS
**KSI = 90.9%**	-	-	-	-	-	-
**Diet selection (g/g)**	**Predicted**					
**Veld hay**	0.655	0.393	0.632	0.557	0.07	NS
**Maize stover**	0.114	0.262	0.147	0.170	0.04	NS
**Sorghum stover**	0.231	0.346	0.222	0.273	0.04	NS
**Diet quality (g/kg DM)**						
**CP**	40.27	41.26	41.77	42.26	1.91	NS
**NDF**	770.91	779.11	761.70	766.53	4.27	NS
**ADF**	450.99	460.44	439.14	444.75	5.18	NS
**Ash**	71.30	76.50	73.68	76.44	4.408	NS
**DMDigestibility**	369^b^	590^a^	428^b^	451^b^	53.00	*
**FR-digestibility**	290	529	430	409	67.78	NS
**KSI = 94.74%**	-	-	-	-	-	-

^1^ KSI, Kulczynski similarity index between the predicted and the observed diet selection and digestibility; CP, crude protein; NDF, neutral detergent fibre; ADF, acid detergent fibre; FR-digestibility, predicted DM digestibility adjusted for incomplete faecal recovery of acid insoluble ash (AIA) marker; ^2^ SEM, standard error of mean; NS, not significantly different (*p* > 0.05); * significantly different (*p* < 0.05); ^a,b^ means with different superscripts within a row differ significantly; (-), omitted values because the KSI is for the overall vales and not for each mean.

**Table 6:** Effect of animal stocking rate on fractional passage rate (h^−1^), mean retention and total tract mean retention time (h).

^1^ Parameter	Stocking Rate (Sheep per Pen)				^2^ Mixed Model	
	1	2	4	8	SEM	Significance
	**Fractional passage rate (h^−1^)**					
**Rumen solids (k_S_)**	0.027	0.031	0.030	0.029	0.005	NS
**Hindgut solids (k_S_)**	0.040	0.042	0.046	0.039	0.009	NS
**Rumen liquids (k_L_)**	0.078	0.054	0.07	0.08	0.367	NS
**Hindgut liquids (k_L_)**	0.115	0.076	1.04	0.101	0.409	NS
	**Mean retention time (h)**					
**Rumensolids**	37.49	41.03	30.88	35.72	7.49	NS
**Hindgutsolids**	30.00	18.80	28.18	27.33	5.93	NS
**Rumenliquids**	16.3	19.7	13.9	13.0	3.37	NS
**Hindgutliquids**	10.4	12.6	13.2	10.1	3.93	NS
	**Total tract mean retention time (h)**					
**Solid fraction MRT**	70.1	62.9	60.7	65.42	11.61	NS
**Liquid fraction MRT**	30.3	34.2	23.8	25.4	6.82	NS

^1^ K_s_, fractional passage rate of solid particles; K_L_, fractional passage rate of liquid particles; MRT, mean retention time; ^2^ SEM, standard error of mean; NS, not significantly different (*p* > 0.05).

**Table 7:** Effect of animal stocking rate on faecal recoveries of internal markers (%).

^1^ Marker	Stocking Rate (Sheep per Pen)				^2^ General Linear Model	
	1	2	4	8	RMSE	Significance
**MADF**	35.5	31.3	32.9	34.9	0.07	NS
**ADL**	49.1	49.3	53.1	47.4	0.15	NS
**AIA**	81.0	118	76.1	76.9	0.41	NS

^1^ MADF, modified acid detergent fibre; ADL, acid detergent lignin; AIA, acid insoluble ash; ^2^ RMSE, root mean square error; NS, not significant (*p* > 0.05).

**Table 8:** Proportion of plant fraction (stem and leaf) in offered diets and the refusals (g/g).

Diet	Proportion of Plant Fraction in Refusals (g/g)									
	Offered		1		2		4		8	
	Stem	Leaf	Stem	Leaf	Stem	Leaf	Stem	Leaf	Stem	Leaf
**MS**	0.713	0.287	0.770	0.230	0.865	0.135	0.773	0.227	0.882	0.118
**SS**	0.701	0.299	0.836	0.164	0.809	0.191	0.766	0.234	0.923	0.077
**VH**	0.606	0.394	0.364	0.636	0.654	0.346	0.285	0.715	0.412	0.588
**SEM**	0.028		0.120		0.052		0.132		0.134	

MS, maize stover; SS, sorghum stover; VH, veld hay; SEM, standard error of mean.
